# The Beck Procedure Revisited: Percutaneous Approach to Myocardial Perfusion Through the Coronary Sinus

**DOI:** 10.1016/j.jaccas.2025.103233

**Published:** 2025-02-26

**Authors:** Khaldoon Alaswad, William O. O’Neill, Asaad Nakhle, Gerald C. Koenig, Brittany S. Fuller, Dee Dee Wang

**Affiliations:** a Henry Ford Hospital, Division of Cardiology, Detroit, Michigan, USA; b Michigan State University College of Human Medicine, East Lansing, Michigan, USA; c Wayne State University, Detroit, Michigan, USA; d Rooney Heart Institute Naples Comprehensive Heath, Naples, Florida, USA

**Keywords:** coronary sinus, in situ bypass, refractory angina, venous arterialization

## Abstract

**Background:**

Many patients live with angina following treatment when all available therapies have failed. Providing retrograde arterial myocardial perfusion using the coronary venous system was performed. The first previously reported percutaneous method might have limited application.

**Early Report Summary:**

We present a first-in-human successful, reproducible, and widely applicable percutaneous procedure using a noncovered coronary stent to connect the left circumflex (LCX) to the coronary sinus (CS) in a patient with previous placement of a CS Reducer.

**Discussion and Novelty:**

Unlike the previous report, our procedure shows for the first time that placement of a noncovered stent between the proximal LCX and the CS did not result in bleeding while effectively relieving the angina. Using a noncovered stent to create a bypass to the CS makes the procedure applicable to more patients with lifestyle-limiting angina.

**Take-Home Message:**

Using the noncovered stent from the LCX to the CS to provide myocardial perfusion might be feasible and safe.

## Graphical Abstract

**Figure:**
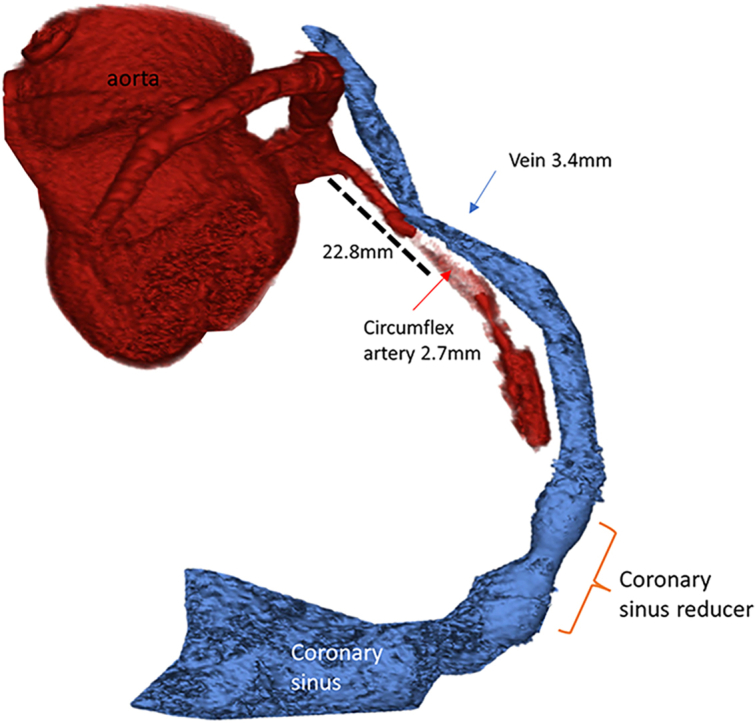

Patients with failed coronary artery bypass grafts (CABGs) and recurrent failure of percutaneous coronary interventions (PCIs) who experience angina refractory to medical therapy have limited therapeutic options. Before the advent of CABG, the Beck procedure involved placement of an arterial to coronary sinus (CS) bypass and surgical narrowing of the CS to relieve angina.^1^ One case report of percutaneous venous arterialization of a branch coronary vein was reported >2 decades ago; this procedure has never been reported again because of the difficulty in selecting branch coronary veins and potential occlusion of the distal coronary artery flow after placement of a tube fistula from the artery to the adjacent vein.^2^

**Take-Home Messages**

• Many patients live with lifestyle-limiting angina after failure with medical therapy and surgical and percutaneous revascularization. Innovations to provide angina relief are underway.
• Percutaneous CS bypass should be explored based on the previous surgical Beck procedure and our presentation.

We present here a first-in-human computed tomography angiography (CTA)-guided use of a noncovered coronary stent to create a percutaneous coronary artery to CS bypass to provide myocardial perfusion in a patient with previous placement of a CS Reducer (Neovasc Inc).

## Case Summary

A 60-year-old male patient with refractory Canadian Cardiovascular Society class III angina despite multiple long-acting antianginal medications and a new reduction in his left ventricular ejection fraction presented for treatment. The patient has a known occluded left internal mammary artery graft to the left anterior descending coronary artery (LAD) and recurrent total occlusion of the LAD stents despite PCIs. He has early-onset coronary artery disease, hyperlipidemia, hypertension, type 2 diabetes, and peripheral artery disease. The patient has received compassionate placement of a CS Reducer, which controlled angina for 4 years before his current presentation.

Percutaneous coronary and graft angiography (Video 1) revealed a patent left main coronary artery, proximal left circumflex (LCX), and the first obtuse marginal branch. The mid LAD stents, the middle LCX, and the proximal right coronary artery were chronically occluded. There was a patent saphenous vein graft to the second obtuse marginal branch. A pharmacologic stress/rest single-photon emission coronary perfusion study showed mixed ischemia and infarction in the LAD territory. According to the heart team, the patient had no other revascularization option. The patient provided informed consent to perform percutaneous venous arterialization.

## Procedures

**Figure 1:**
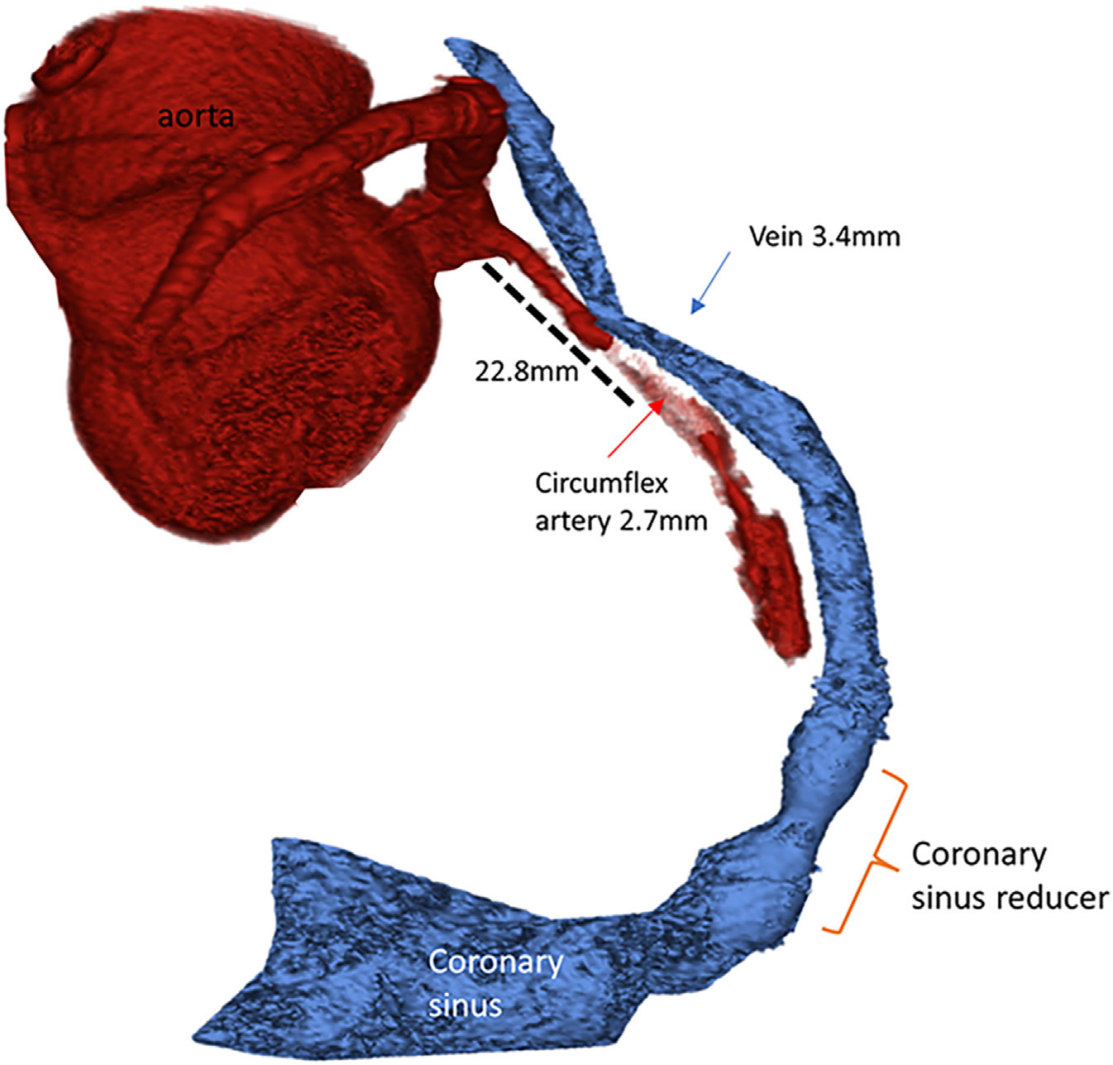
Angio CTO of Coronary Arterial & Venous Systems at Baseline Rendering of the coronary arterial and venous computed tomography angiography.

Pre-procedural coronary arterial and venous mapping was performed with gated contrast-enhanced CTA (Siemens) (Figure 1). The CTA determined the optimal overlap region between the coronary venous and arterial systems at 21.8 mm from the ostium of the LCX. The CTA also provided the best angiographic projections to access the venous system from the arterial side. The anterior greater vein and the LAD did not overlap adequately to provide a safe arterialization option.

An 8-F EBU 4.0 guide catheter (Medtronic) was advanced to the left main coronary artery from a common femoral artery access. A 7-F Attain Command guiding catheter (Medtronic) was advanced to the CS from the right internal jugular vein. Simultaneous arterial and venous coronary angiograms were performed in prespecified projections (Video 2). A microcatheter and coronary guide wire were advanced to the proximal LCX, and a 5-F multipurpose guiding catheter was advanced through the CS Reducer central lumen to the proximal CS. A workhorse coronary guide wire was advanced through the guiding catheter, and a 4 × 40 mm semi-compliant coronary angioplasty balloon was advanced to the CS to overlap with the microcatheter tip in the LCX. The balloon in the CS was inflated, and multiple angiographic projections were reviewed to determine the best overlap with the gear in the proximal LCX.

An Astato 40 wire (Asahi Intecc) failed to enter the CS, using the balloon in the CS as a target. We tried to facilitate entering the CS by dilating the CS with the 4-mm balloon. We exchanged the microcatheter in the LCX for a 6-F Pioneer Plus intravascular ultrasound (IVUS)-based peripheral arterial re-entry catheter (Medtronic). The Pioneer Plus catheter provided IVUS imaging and enough backup to facilitate puncturing with the Astato 40 g wire from the LCX into the CS. The Astato wire was advanced retrogradely into the CS guiding catheter and trapped with a balloon to enable the removal of the Pioneer device without the loss of wire position. The Pioneer device was exchanged for a microcatheter, which was advanced from the LCX to the CS over the Astato wire and then used to exchange the Astato wire for a workhorse coronary wire. The arteriovenous connection was dilated with a 3 × 40 mm coronary angioplasty balloon, followed by deployment of a 3.0 × 38 mm noncovered drug-eluting coronary stent through the track to maintain the atrioventricular bypass.

The patient remained hemodynamically stable throughout the procedure and recovery. The mean pressure in the CS was 17 mm Hg at the baseline and remained unchanged after the procedure. The CS oxygen saturation increased from 42% initially to 78%. Right heart catheterization and transthoracic echocardiogram at the end of the procedure ruled out tamponade and pericardial effusion, respectively.

**Figure 2:**
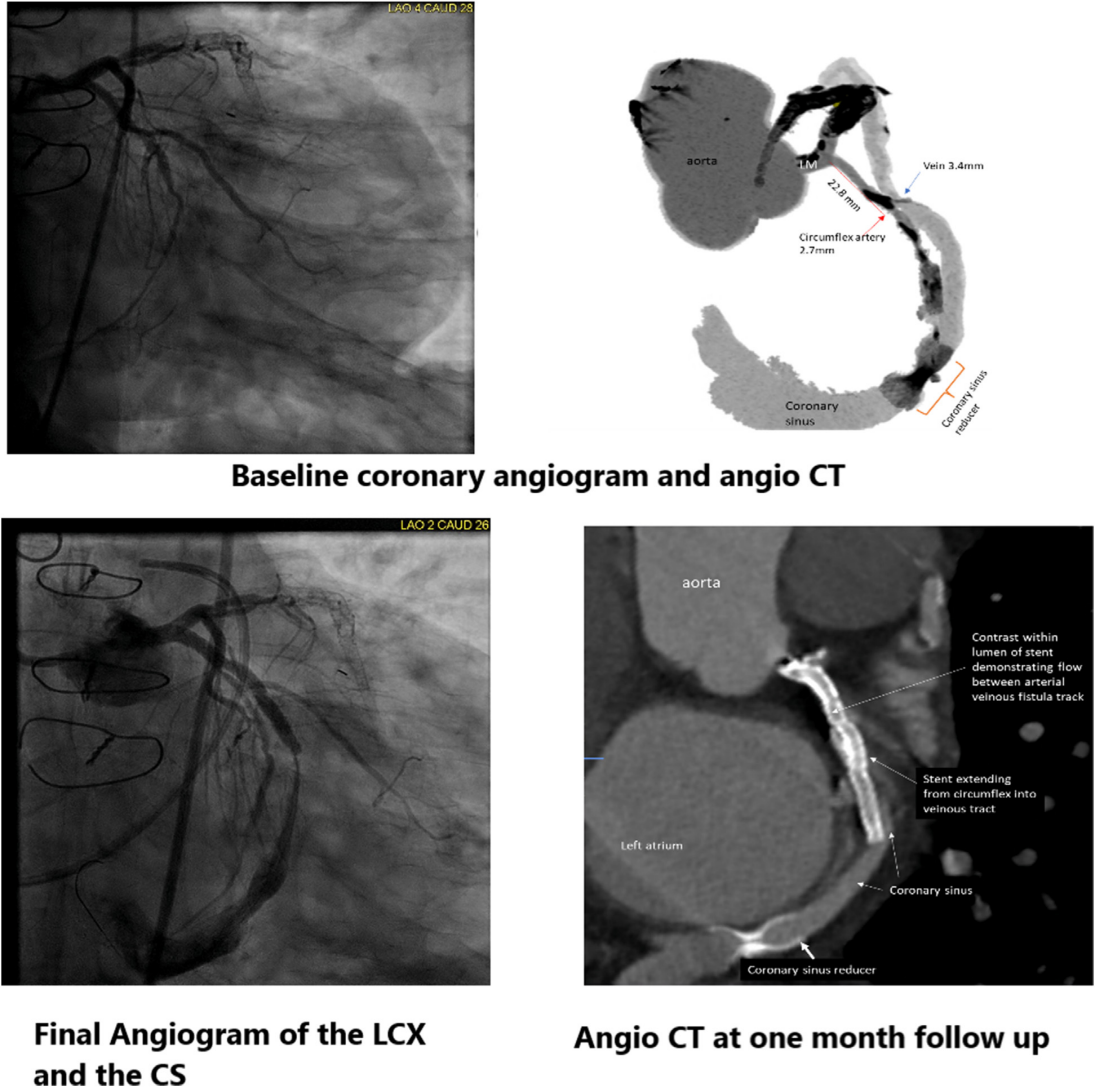
Baseline and Post Procedure Angio CT, and Angiograms (Top) Baseline coronary angiogram and coronary computed tomography angiography (angio CT) depicting the best point of communication to the coronary sinus (CS). (Bottom) Coronary angiogram after the establishment of the arteriovenous bypass and 1-month follow-up angio CT. LCX = left circumflex.

A right heart catheterization and blood saturation performed the next day did not reveal a significant left-to-right shunt, and a transthoracic echocardiogram showed trivial pericardial effusion. The patient was angina-free at the 1-month follow-up, and coronary CTA showed persistent atrioventricular bypass from the LCX to the CS proximal to the CS Reducer (Figure 2). LAD territory perfusion was shown by filling the great anterior vein during the arterial phase. A dobutamine stress echocardiogram revealed no ischemia in the LAD territory. The patient was angina-free at a 1-month follow-up. Three months after the procedure, angiography showed patent LCX to CS bypass.

The patient remained symptom-free until he presented with recurrent angina 1 year after the procedure. Repeat coronary artery and graft angiography showed occlusion of the LCX to CS bypass, and the occlusion extended to the CS Reducer. The bypass and the CS Reducer were recanalized with wire crossing and balloon angioplasty with a cutting balloon at the distal edge of the stent in the CS and 4 mm balloon angioplasty in the rest of the track. IVUS advanced from the LCX to the coronary sinus showed a large, organized clot burden. The patient was discharged on dual antiplatelet therapy and anticoagulation.

## Novelty of the Procedure

Surgical artery to CS bypass with partial CS ligation was first performed by Beck in the 1940s to provide retrograde myocardial revascularization for angina relief.^1^ The first successful percutaneous in situ coronary artery to venous bypass (PICVA) was reported in 2001.^2^ It involved the creation of a tube fistula from the proximal LAD to the anterior interventricular vein and closure of the vein proximally to direct retrograde venous flow to the LAD territory.

Our procedure is significantly different from the PICVA procedure. A percutaneous CS bypass is more likely to be reproducible because the CS and the LCX consistently run parallel and provide a more feasible location to establish the bypass, while finding suitable adjacent branch veins and a patent arterial segment to perform the PICVA procedure is exceptionally challenging. Although the PICVA procedure used a tube fistula to connect the coronary artery and vein, we used a usual noncovered drug-eluting coronary stent, which, unlike a tube fistula or covered stent, will keep the donor artery open to provide distal perfusion. Conversely, the PICVA procedure would have blocked the perfusion to the rest of the LCX artery by the proximal tube fistula. More importantly, we showed for the first time that creating a bypass between the coronary artery and vein with an open struts stent did not result in pericardial bleeding; this can be explained by the low pressure in the venous system, which allowed the blood to be siphoned directly from the artery to the venous system.

This is the first time a CS Reducer has been used with a percutaneous CS bypass to provide retrograde perfusion to the myocardium of an occluded coronary artery, similar to the Beck procedure, without surgery. Percutaneous LCX to CS bypass and CS Reducer placement can replace the branch PICVA procedure and create enough gradient to perfuse the ischemic myocardium in the LAD and the LCX territories.

## Future Direction

Validating the percutaneous LCX to CS bypass with a usual noncovered stent in a larger cohort of patients is essential. Several questions remain to be answered. The scarring from the previous CABG might have prevented bleeding in our patient, although the area around the CS and the proximal LCX rarely develops scarring post-CABG. It is also important to show that the bypass does not result in a steel phenomenon resulting in distal arterial territory hypoperfusion. It needs to be determined if the percutaneous CS bypass without CS Reducer placement will effectively relieve the angina. Our procedure might have been more straightforward with a dedicated coronary IVUS-based technology to direct the penetrating wire from the artery to the vein or vice versa. We speculate that the closure of the CS bypass 1 year later was based on restenosis and thrombosis in the venous system, which raises the question about modification of the procedures or pharmacotherapy to maintain the bypass patency.

## Conclusion**s**

This is the first-in-human demonstration of a safe and effective asynchronous CTA-guided percutaneous CS bypass using the usual noncovered drug-eluting stent. Our procedure shows that percutaneous CS bypass with a CS Reducer relieved the angina. This is confirmed by the fact that occlusion of the CS bypass was associated with recurrent angina.

## Funding Support and Author Disclosures

Dr Alaswad is a consultant and speaker for Boston Scientific and Teleflex; and reports intellectual property in TruVue. Dr Koenig is a consultant for Abbott Vascular and Bard-Becton Dickinson. Dr Fuller is a consultant for Pfizer; and serves on an advisory board committee for Philips. Dr Wang is a consultant for Edwards Lifesciences, Boston Scientific, and Abbott Cardiovascular; and has received institutional research grant support from Boston Scientific. All other authors have reported that they have no relationships relevant to the contents of this paper to disclose.

## Appendix

Video 1 Baseline Coronary and Graft Angiograms

Video 2 Procedure Fluoroscope and Angiograms
